# The Jiangnan diet, a healthy diet pattern for Chinese

**DOI:** 10.1111/1753-0407.13015

**Published:** 2020-01-26

**Authors:** Jiqiu Wang, Xu Lin, Zachary T. Bloomgarden, Guang Ning

**Affiliations:** ^1^ Shanghai National Clinical Research Center for Metabolic Diseases, Department of Endocrinology and Metabolism Ruijin Hospital, Shanghai Jiao Tong University School of Medicine (SJTUSM) Shanghai China; ^2^ Shanghai Institute of Nutrition and Health, Shanghai Institutes for Biological Sciences (SIBS), University of Chinese Academy of Sciences Chinese Academy of Sciences Shanghai China; ^3^ Department of Medicine Icahn School of Medicine at Mount Sinai New York New York

**Keywords:** diet, Jiangnan diet, Mediterranean diet, metabolic syndrome, obesity, 饮食, 江南饮食, 地中海饮食, 代谢综合症, 肥胖

## Abstract

Geographically, the Qinling Mountain—Huai River line divides China into two parts, Northern and Southern. Surprisingly, the line also divides the high prevalence of obesity and metabolic syndrome in Northern China from the low prevalence of Southern China. In past decades, the diet‐center hypothesis has gained much support from the apparent cardiometabolic disease‐protection effect of the Mediterranean diet. Questions include the following: Does the diet pattern explain the disease prevalence difference between two parts with similar genetic background? What kind of diet pattern is suitable for future national diet recommendation for Chinese, as the Mediterranean diet does for the Western countries? Here, we review the main healthy diet components, which the native inhabitants in the Yangtze River Delta region have eaten for several hundreds of years, and refer to this healthy diet as “Southern River (江南)‐style dietary pattern” or “Jiangnan Diet.”

## Graphical abstract

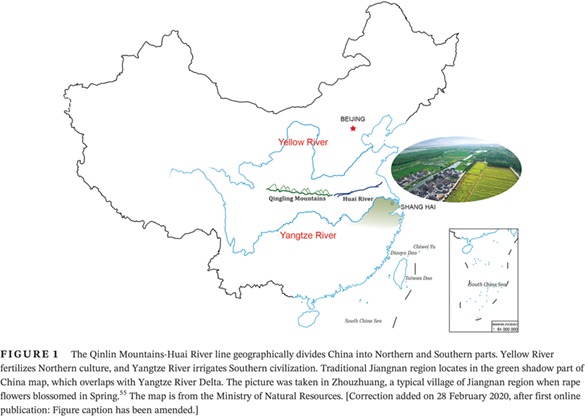

**Highlights**

- To explore the localized “Mediterranean diet” for metabolic disorders prevention in China, we review the main healthy diet components, which the native inhabitants in the Yangtze River Delta region have eaten for a long time, and refer to this healthy diet as “Jiangnan Diet.”

## 摘要

在地理空间上, “秦岭‐淮河”连线将中国版图分为南方和北方两个部分。令人惊讶的是, 这条线也将中国肥胖及代谢性疾病的患病率一分为二:南低北高。过去几十年, 地中海饮食在心血管代谢病中的保护作用被很多研究所证实, 因此代谢性疾病的“饮食中心学说”备受支持。那么, 在相似的遗传背景下, 不同的饮食模式是否能够解释中国南北方代谢性疾病患病率的差异?另外, 以往的饮食研究(包括地中海饮食)多基于欧美人群, 何种健康饮食模式适合今后在国家层面上向全民推荐?在本篇综述中, 我们提炼江南地区几百年来当地居民饮食中的健康组分, 并将其称作“(传统)江南饮食模式" 或"江南饮食”。

1

**Figure 1:**
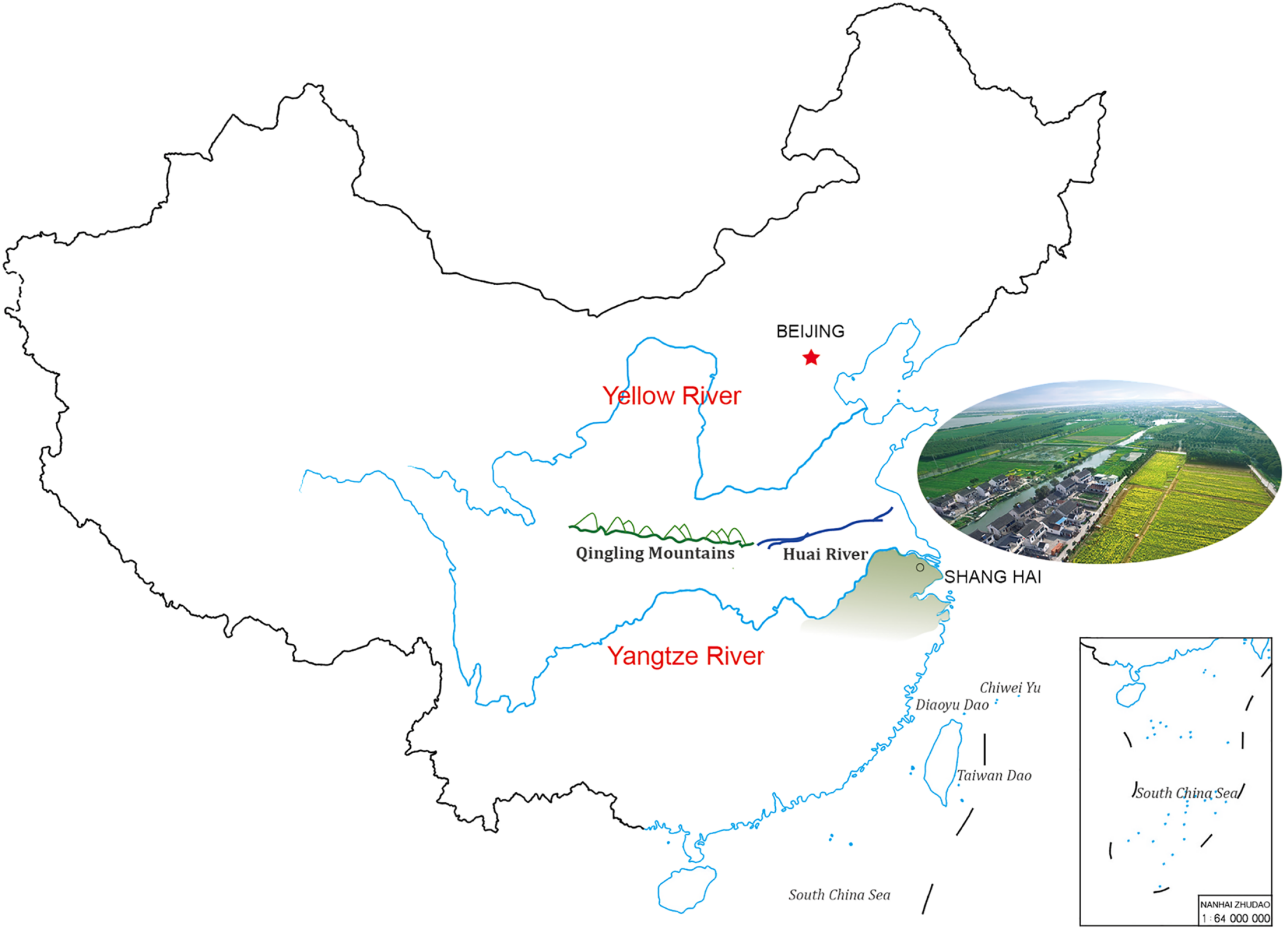
The Qinlin Mountains‐Huai River line geographically divides China into Northern and Southern parts. Yellow River fertilizes Northern culture, and Yangtze River irrigates Southern civilization. Traditional Jiangnan region locates in the green shadow part of China map, which overlaps with Yangtze River Delta. The picture was taken in Zhouzhuang, a typical village of Jiangnan region when rape flowers blossomed in Spring.^55^ The map is from the Ministry of Natural Resources. [Correction added on 28 February 2020, after first online publication: Figure caption has been amended.]

The primitive human civilization originated along the floodplains of large rivers, for example, the Nile River for ancient Egypt, the Tigris and Euphrates Rivers for Mesopotamia, the Indus and Ganges Rivers for ancient India, and the Yellow and Yangtze (Changjiang) Rivers for China, from the west to east.^1^, ^2^ The Yellow and Yangtze Rivers are both derived from the Qinghai‐Tibet plateau, together with the East and South China Seas making a geographic circle protecting China from invasion of other cultures for thousands of years and leading to the development of an intact and continuous culture.^3^ Alluvial flatlands along the Yellow River offered the most fertile soil, readily cultivated, giving birth to the civilization of North China, while the four large lakes and numerous small rivers and canals branching from the Yangtze River in South China gave birth to the Southern China civilization.^4^ One geographical line, marked on the west by the Qinling Mountains and on the east by the Huai River (one of the Yangtze tributaries), divided the whole of China into two parts, Northern and Southern China^5^ (Figure 1). The distinct water system, soil, geographical environment, and climate resulted in two different subcultures, with distinct agriculture, diet, language (accent), stature, and even disease profiles.^6^, ^7^

**Figure 2:**
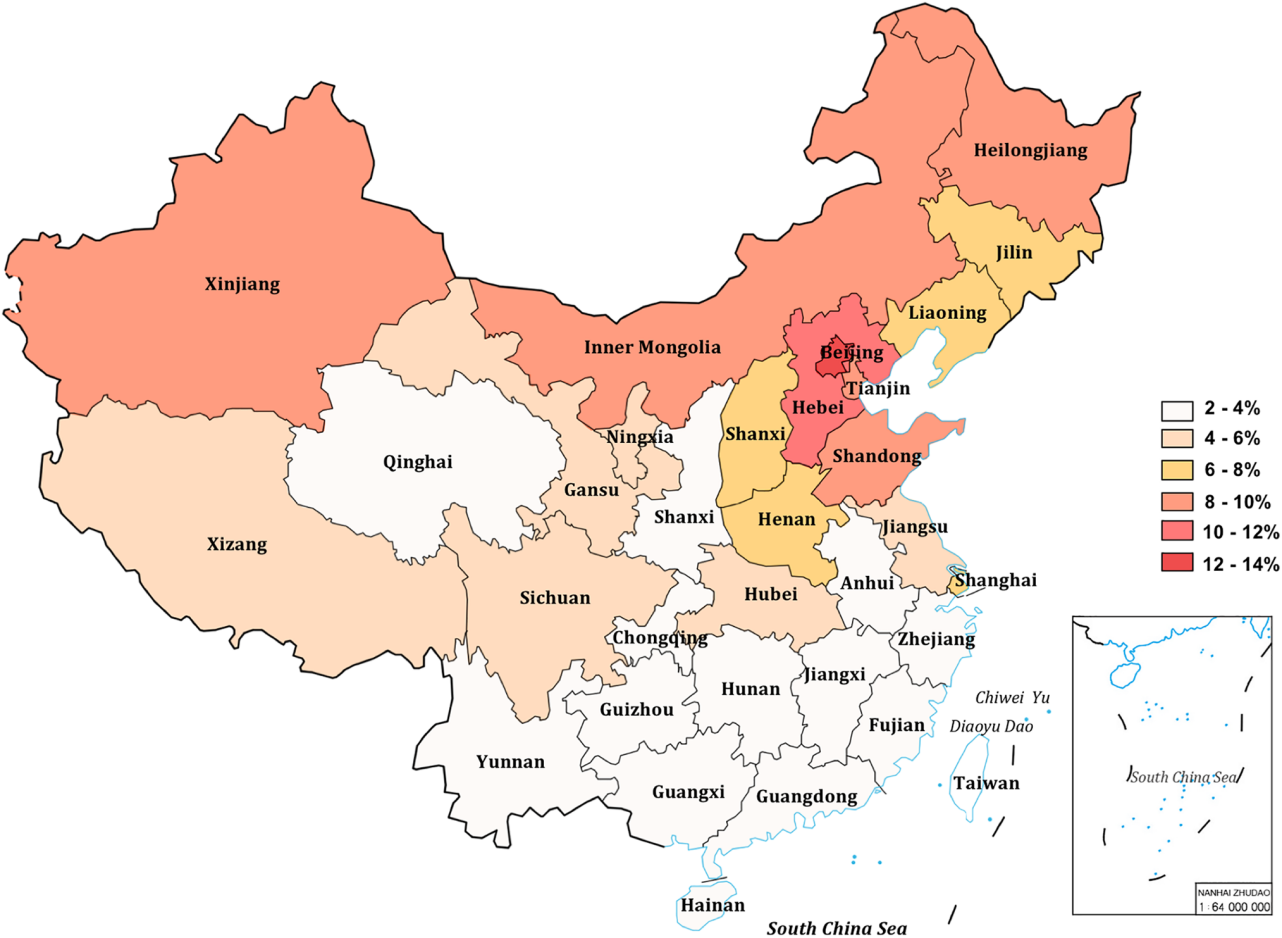
The prevalence of obesity in China in 2010. Obesity criteria are defined with body mass index (BMI) larger than 30 kg/m^2^.^16^ The map is from the Ministry of Natural Resources. [Correction added on 2 April 2020, after first online publication: Figure caption has been amended.]

Over the past four decades, China has experienced tremendous economic and societal development, although parts of China remain underdeveloped. In parallel with this, a huge change in the foods supply and diet components occurred.^8^, ^9^ From the early 1960s to late 1970s, China could not provide sufficient food to majority of its people and rationed grain, meat, and oil. Diet was mainly based on whole grain‐based carbohydrates, in the form of wheat in the North and rice in the South, with limited cooking oil and variaties of vegetables; infrequent consumption of meat, eggs, and dairy products; and little fried food.^10^ From the end of the 1970s, the system of rural reform with household contract responsibility increased the productivity of Chinese farmers^11^; in addition, uses of chemical fertilizers, pesticides, and machinery allowed China to increase its food supply. Recently, energy intake climbed to a high level accompanied with less physical activity, although beginning to decline from the early 1980s.^9^ Meanwhile, the dietary patterns and nutrition composition transited from traditional to more‐Westernized ones.^12^, ^13^ The reduced intake of whole grains and vegetables and the increased consumption of refined grain, red meat, processed meat, sugar‐sweetened beverages, fried food, and unhealthy fat (rich in saturated fat from animal products and tropical plants), led to overwhelming health care burdens despite moderately increasing intake of fruit, nuts, eggs, milk, and unsaturated fatty acids from fish.^9^, ^10^, ^14^ In parallel with economic growth, the prevalence of chronic noncommunicable diseases (NCDs), including obesity, diabetes, cardiovascular diseases (CVD), and cancers, increased considerately.^9^, ^15^, ^16^, ^17^ Taking overweight/obesity for example, the prevalence in Chinese adults rocketed up from about 3.7% in 1980 to about 33.0% in 2010, and mean national adult body mass index increased from 21.1 kg/m^2^ in 1982 to 23.9 kg/m^2^ in 2010‐12.^9^, ^16^, ^18^, ^19^ Surprisingly, the North‐South China line also divided high obesity and metabolic syndrome prevalence in Northern China from low prevalence in Southern China^5^, ^13^, ^16^, ^20^ (Figure 2). Similar disease prevalence distribution is also observed in other NCDs, such as hypertension and CVDs.^15^ The degree of genetic heterogeneity in Northern and Southern Han Chinese is much lower than in Caucasian populations,^21^ and therefore may contribute little to the prevalence discrepancy of the metabolic disorders. Environmental factors, such as climate and air pollution, similarly do not appear to be the dominant contributory factors. We hypothesize that dietary components, styles, or patterns may be one of the major contributors for the NCDs prevalence discrepancy between the two parts of China.^13^, ^22^, ^23^

This diet‐center hypothesis gains further support from the NCD‐protective effect of the Mediterranean diet.^24^, ^25^ Ancel Keys, a physiologist from the University of Minnesota, was considered the first to provide epidemiological evidence of the association of Mediterranean diet and lower CVD mortality with a classic longitudinal cohort study, the Seven Countries Study of Cardiovascular Diseases.^26^ This study began in the late 1950s, recruiting 14 cohorts of middle‐aged men in eight nations of seven countries at baseline. After 5‐10 years follow‐up, Keys and his colleagues found that high saturated fat consumption was associated with CVD mortality, whereas high unsaturated fats appeared protective.^27^, ^28^, ^29^, ^30^, ^31^ High monounsaturated/saturated fat ratios reflected the large intake of olive oil, mainly used in the Mediterranean area. Further follow‐up studies suggest the Mediterranean diet lowered CVD mortality, compared with non‐Mediterranean diet.^29^ Keys himself observed that the Mediterranean diet was a relatively new development.^25^ Based on dietary variation among the countries bordering the Mediterranean, whose populations also vary in genetics, culture, religion, ethnicity, and economic development, the optimal Mediterranean diet formula appears to be characterized by high consumption of monounsaturated/saturated ratio dietary fat (mainly olive oil), vegetables and fruits, legumes, marine fish, and whole grains as nonrefined cereals including bread; moderate intake of wine, milk and dairy products; and low red meat intake. Numerous studies have demonstrated the benefit of components of this diet on health and longevity.^24^, ^32^ Unfortunately, even in rural areas traditionally following the Mediterranean diet, diet patterns are changing, tending to reduce health virtues.^30^ During the past 50 years, consumption of meat and dairy products increased, whereas vegetable intake declined, and the prevalence of NCDs increased in parallel in the Mediterranean region, although still at a lower level than in North America and North Europe.^30^

**Figure 3:**
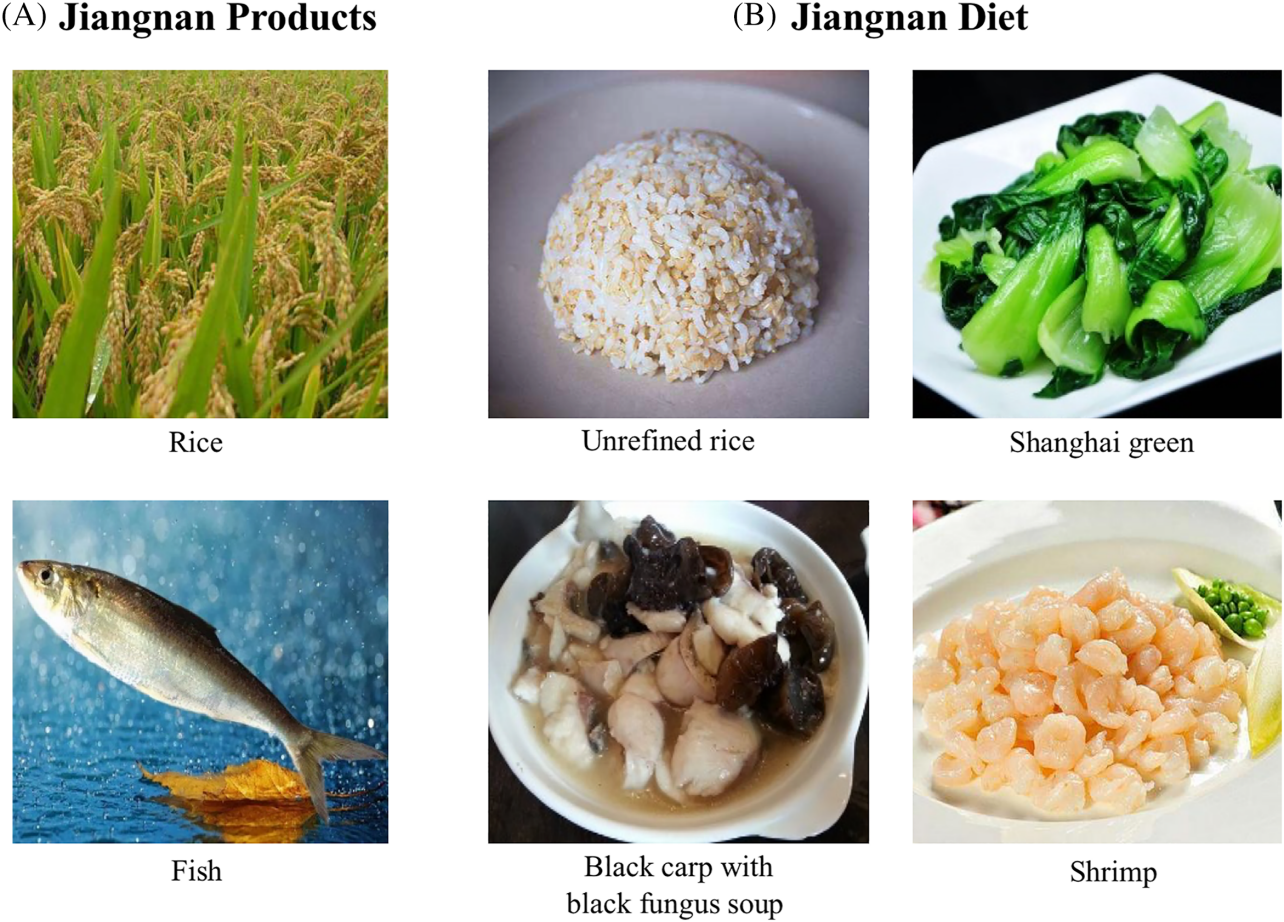
Jiangnan products and a representative dinner composition of Jiangnan diet. A, Yangtze River produced lakes and branched numerous small rivers in the South China, which is suitable for growing rice and fresh water fishes. B, In traditional Jiangnan diet, local inhabitants consume non‐refined rice, adequate vegetables, moderate fresh water fish and shrimp with rapeseed rape oil

What are the implications as far as reduction in NCDs in China, in particular for Northern Chinese? Even in the Spring and Autumn Era (770‐446 B.C.), some diet difference appeared between the northern and southern regions of China.^33^ During the Southern Song Dynasty period (1127‐1279 A.D.), the diet in the North included more salt, and that in the South more sugar, referred to as “Salty North and Sweet South” (南甜北咸). In the subsequent Qing Dynasty period (1636‐1912 A.D.), four major styles of cooking, Guandong (粤菜), Shandong (鲁菜), Sichuan (川菜), and Huaiyang (淮扬菜), were formulated and accepted nationwide.^7^ Nowadays, there is a more complicated Chinese diet system with even more than eight styles of cuisines. Among these southern diet and cooking styles, the Huaiyang, Aihui, and Zhejiang diets share several common features, including high consumption of vegetables and fruits in season, fresh water fish and shrimp, and legumes; moderate consumption of whole‐grain rice, plant oils (mainly rapeseed oil), and red meat; and relatively low consumption of salt, or millet wine. Steaming or boiling in clear soup and lukewarm‐fire frying are the preferred cooking styles (Figure 3). We refer to this healthy diet, typically consumed around the downstream reaches of the Yangtze River, as the Southern River (江南)‐style dietary pattern or “Jiangnan Diet.”

A traditional Chinese saying, “unique water and soil foster its people (一方水土养一方人), expresses the concept that the unique features of a local climate (rain, water, and temperature, etc.) and environment (soil, air, and plants, etc.) always give special ways of living to its inhabitants. Thus, olives from the Mediterranean region have high monounsaturated/saturated fat ratio and CVD benefit. Similarly, rapeseed has grown in the Jiangnan region for thousands of years, and the Southeast Chinese are habituated to use squeezed rapeseed oils, which have a high polyunsaturated/saturated fat ratio (rich in Omega‐6) with a similar protective effect to that of monounsaturated oil in reducing total mortality.^34^, ^35^, ^36^ Additionally, they also consume relatively high Omega‐3 unsaturated fatty acids from fish. However, Northern Chinese maintain higher consumption of wheat flour, starch, tubers, and other cereals, and lower consumption of vegetables and fruits, and more animal foods, with lower CVD benefit^13^; meanwhile, they like to cook with boiling oil, which usually ruins the good mono‐ and polyunsaturated fat and turns it into saturated or trans fat. Given the increasing proportion of dietary fat among the total energy intake in China, the outcomes of the fat source and its cooking ways need more investigation. There are at least three obstacles to widespread olive oil usage in ordinary Chinese people: cost, taste and cooking with relatively high temperature, so that use of rapeseed, sunflower or soybean oils after steaming or boiling could be associated with healthy fat characteristics in the Jiangnan Diet.

The Jiangnan region has sufficient rainfall, a complicated water system, fertile plains, and a short winter, producing large varieties of vegetables and fruits around seasons, as well as plenty of fresh water fishes, shrimps, crabs and ducklings. The typical water villages are like Zhouzhuang in Suzhou, Jiangsu Province, Hongcun in Huangshan, Anhui Province, and Wuzhen in Shaoxing, Zhejiang Province, reserve the layouts of hundreds or even thousands of years (Figure 1). The plentiful supply of food resource here feed the large and high‐density population of the world, meanwhile, Jiangnan inhabitants have a higher average life expectancy than that of other regions.^20^, ^37^ Their daily intake of abundant vegetables and moderate fruits is the one of common features shared by the Mediterranean and Jiangnan diets,^38^ whereas Jiangnan region has more dark green leafy vegetables. Both diets also take more high‐quality white meat rather than red meat in comparison with the diet of the Northern Chinese.^39^, ^40^ One of the major differences is that the Jiangnan diet contains larger portion of Doufu and other soybean products and smaller portion of milk and other dairy products than the Mediterranean diet. The Jiangnan diet has less or no smoked, salty, or pickled vegetables than that consumed in Northern China, especially during the winter.^41^ The Mediterranean inhabitants included whole grains as the main carbohydrate source years ago, as was also used in the Jiangnan area before the 1990s.^9^ The distinction is that the former diet included nonrefined barley and wheat, the latter nonrefined rice. Currently, optimal diet approaches both for the Mediterranean and Jiangnan regions include more traditional whole‐grain and less refined flour or rice.^42^, ^43^ Unfortunately, low whole‐grain intake and high refined grain intake have been the major dietary risk factors for diabetes in China nowadays.^44^ The Jiangnan natives do not take so much grape wine as the Mediterranean do, but males there take little millet wine instead. Traditionally, the portion of each dish in Jiangnan region is small and people usually enjoy more varieties of dishes with the same or even less energy intake compared with their northern counterparts. Despite of numerous other differences in dietary composition between the two regions, we here conclude the main commonalities and differences that are major risk or protective factors of NCDs.

Is the Mediterranean diet the best pattern for Western people? Is it also the best diet in the general population? Is it still the best choice for East Asian people?^45^, ^46^ Is it preferable to the Jiangnan diet for Southern Chinese? Notably, we have little evidence for the comparison of these diets with convincing evidence‐based data, that is, in large‐scale longitudinal cohort studies and in randomized control dietary or feeding intervention trials with incident disease as outcome.^24^, ^47^, ^48^, ^49^ Several studies conducted in non‐Mediterranean western areas have found difficulties in achieving high compliance to follow Mediterranean diet except with intensive intervention.^50^ It is almost impossible to perform a long‐term feeding trial in a large‐scale population. We can investigate the effects of one component addition or deletion on health or disease incidence,^24^ but it will be much more difficult to compare different dietary patterns.^51^ Close adherence only to one diet seems to betray the nature of humans' feeding behavior and needs forceful will. The precise record and analysis of daily calorie intake and nutrition compositions are other technical barriers until now. Other confounding factors including age, gender, ethnic and genetic differences, physical activity, smoking and drinking habits, basal metabolic rate, stress and mental status, and even gut microbes also have interaction with diet and should be well controlled and well recorded.^52^ It will be a breakthrough to resolve these difficulties and provide some evidence about which diet produces more benefits on health. It could be concluded that both the traditional Mediterranean diet and the traditional Jiangnan diet are close to the Healthy Plate Recommendation by Harvard Nutrition.^53^ Of course, when we talk about the health benefits of one type of diet, it should be taken account whether it is sufficient, sustainable, cost‐effective, and causes no damage to the environment.

The Da Qing Study provided the first solid evidence that lifestyle intervention, including diet and exercise, significantly reduces the incidence of diabetes in a glucose intolerant population with subsequent improvement in all‐cause mortality.^9^, ^54^ If we tend to identify a relatively comparable healthy diet as the Mediterranean diet, for example, the Jiangnan Diet, what should we learn from Da Qing Study? To lower the rising prevalence of NCDs at present and following decades, we need to act now and identify the major healthy and unhealthy components of different regional diets and collect enough evidence from large‐scale prospective cohort studies, intervention trials, and biological experiments with collaborations of public health investigators, policymakers, administrators, physicians, nutritionists, basic scientists, and even meal providers. Therefore, we can recommend healthy dietary patterns, effective and applicable in the prevention of NCDs, sustainable in terms of cost and taste, both for Jiangnan natives and those from other regions. The healthy Jiangnan diet could be as one of the excellent choices.

## DISCLOSURE

The authors declare no competing interests.

## References

[1] Macklin MG , Lewin J . The rivers of civilization. Quat Sci Rev. 2015;114:228‐244.

[2] Yevjevich V . Water and civilization. Water Int. 1992;17(4):163‐171.

[3] Fan J , Yu H . Nature protection and human development in the Selincuo region: conflict resolution. Sci Bull. 2019;64(7):425‐427. 10.1016/j.scib.2019.03.014 36659790

[4] Zhao Z . New Archaeobotanic data for the study of the origins of agriculture in China. Curr Anthropol. 2011;52(S4):S295‐S306.

[5] Ji CY , Cheng TO . Prevalence and geographic distribution of childhood obesity in China in 2005. Int J Cardiol. 2008;131(1):1‐8. 18765165 10.1016/j.ijcard.2008.05.078

[6] Song F , Cho MS . Geography of food consumption patterns between south and North China. Foods. 2017;6(5):1‐13. 10.3390/foods6050034 PMC5447910 28475146

[7] Chen CK . The culture of Chinese diet: regional differentiation and developing trends. Acta Geogr Sin. 1994;49(3):226‐235.

[8] Ge K . The transition of Chinese dietary guidelines and the food guide pagoda. Asia Pac J Clin Nutr. 2011;20(3):439‐446. 21859664

[9] Gong Q , Zhang P , Wang J , et al. Morbidity and mortality after lifestyle intervention for people with impaired glucose tolerance: 30‐year results of the Da Qing diabetes prevention outcome study. Lancet Diabetes Endocrinol. 2019;7(6):452‐461. 31036503 10.1016/S2213-8587(19)30093-2 PMC8172050

[10] Lam HM , Remais J , Fung MC , Xu L , Sun SSM . Food supply and food safety issues in China. Lancet. 2013;381(9882):2044‐2053. 23746904 10.1016/S0140-6736(13)60776-X PMC3888022

[11] Krusekopf CC . Diversity in land‐tenure arrangements under the household responsibility system in China. China Econ Rev. 2002;13(2–3):297‐312.

[12] Yu D , Zhang X , Xiang YB , et al. Adherence to dietary guidelines and mortality: a report from prospective cohort studies of 134,000 Chinese adults in urban Shanghai. Am J Clin Nutr. 2014;100(2):693‐700. 24944055 10.3945/ajcn.113.079194 PMC4095665

[13] He Y , Ma G , Zhai F , et al. Dietary patterns and glucose tolerance abnormalities in Chinese adults. Diabetes Care. 2009;32(11):1972‐1976. 19675202 10.2337/dc09-0714 PMC2768212

[14] Bi Y , Jiang Y , He J , et al. Status of cardiovascular health in Chinese adults. J Am Coll Cardiol. 2015;65(10):1013‐1025. 25766949 10.1016/j.jacc.2014.12.044

[15] Chen W , Zheng R , Baade PD , et al. Cancer statistics in China, 2015. CA Cancer J Clin. 2016;66(2):115‐132. 26808342 10.3322/caac.21338

[16] Xu Y , Wang L , He J , et al. Prevalence and control of diabetes in Chinese adults. JAMA. 2013;310(9):948‐959. 24002281 10.1001/jama.2013.168118

[17] Yang ZJ , Liu J , Ge JP , et al. Prevalence of cardiovascular disease risk factor in the Chinese population: the 2007‐2008 China National Diabetes and metabolic disorders study. Eur Heart J. 2012;33(2):213‐220. 21719451 10.1093/eurheartj/ehr205

[18] Abarca‐Gómez L , Abdeen ZA , Hamid ZA , et al. Worldwide trends in body‐mass index, underweight, overweight, and obesity from 1975 to 2016: a pooled analysis of 2416 population‐based measurement studies in 128·9 million children, adolescents, and adults. The Lancet. 2017;390(10113):2627‐2642. 10.1016/S0140-6736(17)32129-3 PMC5735219 29029897

[19] Ma G , Li Y , Wu Y , et al. The prevalence of body overweight and obesity and its changes among Chinese people during 1992 to 2002. Zhonghua Yu Fang Yi Xue Za Zhi. 2005;39(5):311‐315. 16266539

[20] Gu D , Reynolds K , Wu X , et al. Prevalence of the metabolic syndrome and overweight among adults in China. Lancet. 2005;365(9468):1398‐1405. 15836888 10.1016/S0140-6736(05)66375-1

[21] Chen J , Zheng H , Bei J‐X , et al. Genetic structure of the Han Chinese population revealed by genome‐wide SNP variation. Am J Hum Genet. 2009;85(6):775‐785. 19944401 10.1016/j.ajhg.2009.10.016 PMC2790583

[22] Wang D , He Y , Li Y , et al. Dietary patterns and hypertension among Chinese adults: a nationally representative cross‐sectional study. BMC Public Health. 2011;11(1):925. 22168909 10.1186/1471-2458-11-925 PMC3299712

[23] Yap IK , Brown IJ , Chan Q , et al. Metabolome‐wide association study identifies multiple biomarkers that discriminate north and south Chinese populations at differing risks of cardiovascular disease: INTERMAP study. J Proteome Res. 2010;9(12):6647‐6654. 20853909 10.1021/pr100798r PMC3117148

[24] Estruch R , Ros E , Salas‐Salvadó J , et al. Primary prevention of cardiovascular disease with a Mediterranean diet supplemented with extra‐virgin olive oil or nuts. N Engl J Med. 2018;378(25):e34. 29897866 10.1056/NEJMoa1800389

[25] Keys A . Mediterranean diet and public health: personal reflections. Am J Clin Nutr. 1995;61(6):1321S‐1323S. 7754982 10.1093/ajcn/61.6.1321S

[26] Keys A . Coronary heart disease in seven countries. Circulation. 1970;41(1):186‐195. 5442775

[27] Blackburn H . Invited commentary: 30‐year perspective on the seven countries study. Am J Epidemiol. 2017;185(11):1143‐1147. 28535176 10.1093/aje/kwx071

[28] Fidanza F , Alberti A , Lanti M , Menotti A . Mediterranean adequacy index: correlation with 25‐year mortality from coronary heart disease in the seven countries study. Nutr Metab Cardiovasc Dis. 2004;14(5):254‐258. 15673059 10.1016/s0939-4753(04)80052-8

[29] Keys A , Mienotti A , Karvonen MJ , et al. The diet and 15‐year death rate in the seven countries study. Am J Epidemiol. 1986;124(6):903‐915. 3776973 10.1093/oxfordjournals.aje.a114480

[30] Menotti A , Puddu P . How the seven countries study contributed to the definition and development of the Mediterranean diet concept: a 50‐year journey. Nutr Metab Cardiovasc Dis. 2015;25(3):245‐252. 25650160 10.1016/j.numecd.2014.12.001

[31] Menotti A , Puddu P , Lanti M , Maiani G , Catasta G , Fidanza AA . Lifestyle habits and mortality from all and specific causes of death: 40‐year follow‐up in the Italian rural areas of the seven countries study. J Nutr Health Aging. 2014;18(3):314‐321.2462676110.1007/s12603-013-0392-1

[32] Hu FB , Willett WC . Optimal diets for prevention of coronary heart disease. JAMA. 2002;288(20):2569‐2578. 12444864 10.1001/jama.288.20.2569

[33] Wang XP , Zhou AD . Geographical environment and southern‐Nothern diet culture of Xian‐Qin. Diet Cult Res. 2006;19(3):85‐90.

[34] Wang DD , Li Y , Chiuve SE , et al. Association of specific dietary fats with total and cause‐specific mortality. JAMA Intern Med. 2016;176(8):1134‐1145. 27379574 10.1001/jamainternmed.2016.2417 PMC5123772

[35] Jiao J , Liu G , Shin HJ , et al. Dietary fats and mortality among patients with type 2 diabetes: analysis in two population based cohort studies. BMJ. 2019;366 :l4009. 31266749 10.1136/bmj.l4009 PMC6603712

[36] Zong G , Liu G , Willett WC , et al. Associations between linoleic acid intake and incident type 2 diabetes among U.S. men and women. Diabetes Care. 2019;42(8):1406‐1413. 31182488 10.2337/dc19-0412 PMC6647042

[37] Zhou M , Wang H , Zeng X , et al. Mortality, morbidity, and risk factors in China and its provinces, 1990–2017: a systematic analysis for the global burden of disease study 2017. Lancet. 2019;394:1145‐1158. 31248666 10.1016/S0140-6736(19)30427-1 PMC6891889

[38] Drewnowski A . New metrics of affordable nutrition: which vegetables provide most nutrients for least cost? J Acad Nutr Diet. 2013;113(9):1182‐1187. 23714199 10.1016/j.jand.2013.03.015

[39] Pan A , Sun Q , Bernstein AM , et al. Red meat consumption and risk of type 2 diabetes: 3 cohorts of US adults and an updated meta‐analysis. Am J Clin Nutr. 2011;94(4):1088‐1096. 21831992 10.3945/ajcn.111.018978 PMC3173026

[40] He Y , Li Y , Yang X , et al. The dietary transition and its association with cardiometabolic mortality among Chinese adults, 1982–2012: a cross‐sectional population‐based study. Lancet Diab Endocrinol. 2019;7:540‐548. 10.1016/S2213-8587(19)30152-4 PMC7269053 31085143

[41] Lu SH , Camus AM , Tomatis L , Bartsch H . Mutagenicity of extracts of pickled vegetables collected in Linhsien County, a high‐incidence area for esophageal cancer in northern China. J Natl Cancer Inst. 1981;66(1):33‐36. 7005503

[42] Wu H , Pan A , Yu Z , et al. Lifestyle counseling and supplementation with flaxseed or walnuts influence the management of metabolic syndrome. J Nutr. 2010;140(11):1937‐1942. 20826632 10.3945/jn.110.126300 PMC3361016

[43] Zhang G , Malik VS , Pan A , et al. Substituting brown rice for white rice to lower diabetes risk: a focus‐group study in Chinese adults. J Am Diet Assoc. 2010;110(8):1216‐1221. 20656097 10.1016/j.jada.2010.05.004

[44] Li Y , Wang DD , Ley SH , et al. Time trends of dietary and lifestyle factors and their potential impact on diabetes burden in China. Diabetes Care. 2017;40(12):1685‐1694. 29046327 10.2337/dc17-0571 PMC5862128

[45] Mancini JG , Filion KB , Atallah R , Eisenberg MJ . Systematic review of the Mediterranean diet for long‐term weight loss. Am J Med. 2016;129(4):407‐415. e404. 26721635 10.1016/j.amjmed.2015.11.028

[46] Thom G , Lean M . Is there an optimal diet for weight management and metabolic health? Gastroenterology. 2017;152(7):1739‐1751. 28214525 10.1053/j.gastro.2017.01.056

[47] Astrup A , Larsen TM , Harper A . Atkins and other low‐carbohydrate diets: hoax or an effective tool for weight loss? Lancet. 2004;364(9437):897‐899. 15351198 10.1016/S0140-6736(04)16986-9

[48] Foster GD , Wyatt HR , Hill JO , et al. A randomized trial of a low‐carbohydrate diet for obesity. N Engl J Med. 2003;348(21):2082‐2090. 12761365 10.1056/NEJMoa022207

[49] Samaha FF , Iqbal N , Seshadri P , et al. A low‐carbohydrate as compared with a low‐fat diet in severe obesity. N Engl J Med. 2003;348(21):2074‐2081. 12761364 10.1056/NEJMoa022637

[50] Murphy KJ , Parletta N . Implementing a Mediterranean‐style diet outside the Mediterranean region. Curr Atheroscler Rep. 2018;20(6):28. 29728772 10.1007/s11883-018-0732-z

[51] Gardner CD , Trepanowski JF , Del Gobbo LC , et al. Effect of low‐fat vs low‐carbohydrate diet on 12‐month weight loss in overweight adults and the association with genotype pattern or insulin secretion: the DIETFITS randomized clinical trial. JAMA. 2018;319(7):667‐679. 29466592 10.1001/jama.2018.0245 PMC5839290

[52] Gambhir SS , Ge TJ , Vermesh O , Spitler R . Toward achieving precision health. Sci Transl Med. 2018;10(430):eaao3612. 29491186 10.1126/scitranslmed.aao3612 PMC5985668

[53] Swartzberg J , Margen S . Eat, drink, and be healthy: the Harvard Medical School guide to healthy eating. Am J Epidemiol. 2001;154(12):1160‐1160.

[54] Pan XR , Li GW , Hu YH , et al. Effects of diet and exercise in preventing NIDDM in people with impaired glucose tolerance: the Da Qing IGT and diabetes study. Diabetes Care. 1997;20(4):537‐544. 9096977 10.2337/diacare.20.4.537

[55] http://www.zhouzhuang.net/index.php?c=article&a=type&tid=95&season=%E6%98%A5%E5%AD%A3.

